# Perspective matters in recovery: the views of persons with severe mental illness, family and mental health professionals on collaboration during recovery, a qualitative study

**DOI:** 10.1186/s12888-024-06198-w

**Published:** 2024-11-14

**Authors:** Thijs J. Burger, Robin M. van Eck, Marjolein Lachmeijer, Kimriek R. G. de Wilde-Schutten, Mette Lansen, Carola van Alphen, Niek van Haasteren, Karin Groen, Frederike Schirmbeck, Astrid Vellinga, Martijn J. Kikkert, Jack Dekker, Lieuwe de Haan, Mariken B. de Koning

**Affiliations:** 1 Arkin, Institute for Mental Health, Afdeling Onderzoek, Klaprozenweg 111, Amsterdam, 1033NN The Netherlands; 2 Department of Psychiatry, Amsterdam University Medical Centre, Amsterdam, The Netherlands; 3 Anoiksis, Association for Persons With Psychosis Susceptibility, Utrecht, The Netherlands; 4 Ypsilon, Association for Family and Network Members of Persons With Psychosis Susceptibility, Den Haag, The Netherlands; 5 Department of Public Mental Health, Central Institute of Mental Health, Medical Faculty Mannheim, Heidelberg University, Mannheim, Germany; 6 Department of Clinical Psychology, Vrije Universiteit, Amsterdam, The Netherlands

**Keywords:** Severe mental illness, Psychosis, Insight, Network approach, Family involvement, Long-term care, Psychiatric rehabilitation, Community mental health

## Abstract

**Background:**

Recovery from severe mental illness, including psychosis has been described as a personal and unique process, but it rarely is a journey undertaken without profound influences of significant others (family, mental health professionals). Diverging perspectives between persons with severe mental illness, family and professionals are frequent during the recovery process, notably in psychotic disorders. We aimed to explore processes of collaboration during recovery, to inform recovery supporting practices.

**Methods:**

Current qualitative study had a participatory design and was set within long-term mental healthcare for severe mental illness. We conducted semi-structured interviews and focus groups with persons with severe mental illness (most had a history of psychosis), family and professionals on their mutual contact during recovery. Using reflexive thematic analysis, we developed themes representing processes of collaboration during recovery.

**Results:**

We described roles persons with severe mental illness, family and professionals attribute to each other in mutually influential terms of unconditional and meaningful contact (which takes time to establish) and problem-oriented aspects. Secondly, experienced differences over problem definition, “needing help” and consequently over the role parties attribute to one another, may result in negative interactions, in the area of having expectations; (not) informing; (not) having agency to change; experiencing (dis)agreement or struggle.

**Conclusions:**

unconditional, meaningful contact and knowing each other’s perspective are important to fruitful interaction in a triad when perspectives on mental health problems diverge. Relationally centered and process oriented care with continuity of family and professionals involved are needed to advance recovery in severe mental illness, especially psychosis.

**Supplementary Information:**

The online version contains supplementary material available at 10.1186/s12888-024-06198-w.

## Background

Personal recovery (referred to hereafter as “recovery”) from severe mental illness has been conceptualized as a deeply personal and unique, non-linear process of change, to which developments in connectedness, hope, identity, meaning of life and empowerment contribute [1, 2]. However, it rarely is a journey undertaken without profound influences from significant others, from both social (family, friends, important others, referred to as “family” hereafter) and professional networks (involving an array of mental health professionals, referred to as “professionals” hereafter) [3, 4]. These influences may have both positive and negative aspects. For example, family may react to the burden of mental illness with intensive involvement or by distancing themselves, and professionals use care paradigms, such as the medical model, to provide care [5]. These modes may, or may not, align to individual recovery needs of someone with severe mental illness [6–8].

Differences in view (“diverging perspectives”) on what is going on and what is needed between persons with severe mental illness, family and professionals are considered a central characteristic of persisting severe mental illness [9, 10]. Persons with severe mental illness, especially during psychosis, may experience a pressing reality unseen and/or misunderstood by others [11, 12]. Family may struggle to address problems they (for)see [13]. Professionals may conceptualize the situation as lack of insight in having a mental illness, metacognitive deficits, or denial as a defence to disturbing thoughts or behaviour [10, 14, 15].

Diverging perspectives may be viewed as a root of problems or as a resource for change. Interventions aimed at involving family or enhancing collaboration between persons with severe mental illness, family and professionals result in better outcomes, such as reduced caregiver burden, or lower relapse and readmission rates [16–18]. The recovery process itself may also benefit [19]. As Seikkula and Arnkil put it, “there are surprising and unexpected resources to be found through thinking together” [20]. In other words, mutual involvement of professionals, persons with severe mental illness *and* their family holds the potential to a larger problem-solving capacity.

Mental Health systems around the world have prioritised working together with families to facilitate recovery from severe mental illness [21–23]. However, research also shows that parties involved in recovery experience barriers in involving families, related to the individual or relational level (e.g. relational difficulties in families), organisation of care (e.g. priority setting) and culture paradigm (e.g. stigma) [24]. So, the collaboration between persons with severe mental illness, family and mental health professionals during recovery (referred to hereafter as a “triad”) comes with both opportunities and challenges.

A better understanding of the processes at play between persons with severe mental illness, family and professionals during recovery may facilitate collaboration in a triad. In this participative qualitative study, we performed a bottom-up exploration of the experience of collaboration during recovery. By bottom up, we mean that we took the subjective experience of persons with mental illness, family and professionals as a starting point and focus. We chose this bottom up approach because we wanted to give persons with severe mental illness and their family a voice on collaboration in mental healthcare. We chose the context of long term mental healthcare for persons with severe mental illness (most have a history of psychosis or enduring psychotic experiences), where both mental health issues and mutual contact between the three parties are long-term by definition. Focusing on relational aspects of the accounts of persons with severe mental illness, family and professionals, we developed themes representing processes of collaboration for recovery in a triad, aiming to help a collaborative recovery supporting practice.

## Methods

### Study design, aim and setting

We performed a participative qualitative study using a reflexive thematic analysis of interviews and focus groups according to Braun and Clarke [25, 26]. We aimed to develop a bottom up understanding of collaboration for recovery, based on subjective experiences of persons with severe mental illness, family or important others (“family”) and mental healthcare workers (“professionals”) on their contact with the other parties during the recovery process.

Our research was set in the context of long-term mental healthcare practice in Amsterdam, the Netherlands, which is predominantly, but not exclusively, aimed at persons with a severe mental illness who have a psychotic disorder [27, 28]. Persons with mental illness in this type of care have the following general attributes: a mental illness with a protracted character, with severe impairments in social domains, and the need for prolonged and coordinated contact with mental health networks focused on multiple areas of life [29]. Specifically, we focused on persons receiving assertive community based treatment or treatment in long-term inpatient mental health rehabilitation units. Both types of care are aimed at people with a severe mental illness who (regularly) need an assertive approach by mental health professionals, to engage effectively with mental health services. People often remain in care of these type of services for years, if not decades. By including the long-term inpatient mental health rehabilitation setting, we specifically sought to include the otherwise marginalized voice of people with the most complex mental health needs and their family, whose needs could not be adequately addressed in a community mental health setting [31, 32]. Prolonged harmful situations for themselves or others often have resulted, including loss of housing [27].

We made use of semi-structured interviews to collect rich, in-depth information from the perspective of persons with severe mental illness, family and professionals on their experience in the triad during recovery. After initial analysis, we held focus group meetings to enrich our material by including peer-to-peer and between-perspective interactions, and triangulate our initial findings. Four “homogeneous” focus groups included participants of one perspective per group, and one “mixed” focus group included all three perspectives.

The study had a participatory design. The research aim and methods were developed in a project group in which the perspectives of persons with severe mental illness, their family and professionals were represented. Individual interviews and focus groups were conducted by pairs of a researcher and an expert by experience from a personal or family perspective, to facilitate rapport with the respondent based on similar experiences. Indeed, some respondents explicitly stated that the input of the expert by experience was essential for their openness, and our impression was that this often was the case. Lastly, the project group reviewed preliminary results and the draft manuscript.

### Recruitment and ethical considerations

Recruitment took place within Arkin, a large mental healthcare institution in Amsterdam, The Netherlands, between July 2018 and March 2020. We recruited persons with severe mental illness receiving assertive community-based treatment, or treatment in long-term inpatient mental health rehabilitation units, via their mental health professionals and used snowball-sampling to recruit their family and professionals. Additionally, family members were approached directly through family meetings regularly organised by the institution. Within this convenience sample, we purposively sought for variety in sex and setting (long-term assertive community based/long-term inpatient service). For families and professionals, we additionally sought for heterogeneity in role (e.g. sibling or parent; nurse or psychiatrist). Interview and homogeneous focus group participants did not overlap. Mixed focus group participants were recruited from interview- and homogeneous focus group participants, and did not include persons who were part of the same triad. Inclusion criteria were age of 18 years or older, ability to give informed consent, and proficiency in Dutch.

The study protocol was reviewed by the institutional review board of the Vrije Universiteit Medical Centre (reference 2018/196), and was granted an exemption from approval based on the fact that participants in the study were not subject to procedures or interventions, or required to follow rules of behavior. All respondents gave written informed consent to participate. When patients had a court-appointed legal representative, which especially occurred in respondents living in long-term inpatient services, we additionally obtained their informed consent.

### Interviews and focus groups with service users, their families and health professionals

The recovery-oriented interview propagated by (among others) van Os et al. served as a basis for the interview (what happened, strengths, weaknesses, future hopes or goals), facilitating the respondents in telling a personal, detailed, in-depth narrative on their (role in) recovery from severe mental illness [33]. The topic guide was pilot tested among interviewers and co-interviewers and probed for more detail when dealings with the other parties within the recovery triad were touched upon (see additional material: 1. topic guide). We invited persons with severe mental illness and families to reflect on dealings with parties currently or formerly part of the triad. Similarly, we allowed professionals to reflect on the index-case, but also on other cases they had been involved in. Interviews were held at home or at an institution location according to respondent’s preferences by pairs of a researcher trained in qualitative research (KW, MdK, ML or TB) and an expert by experience, who both identified as such. All interviews were voice-recorded and transcribed verbatim. Field notes were audio-recorded after the interview by the interview team. As a member check of what we took from the interviews, we shared a narrative summary of the interview with the respondent afterwards and asked for feedback.

In the focus groups, after a round of introductions, we asked participants to write or draw up a collage of what they hoped for the future for themselves and to others in the triad, and who helped and/or hindered in achieving this. Then, we started an exchange using their collages as a starting point, and encouraged dialogue when the conversation focused on taking on helping and hindering roles and interactions within triads. Focus groups were held at an institution location and moderated by MdK and an expert by experience, were voice-recorded, and transcribed verbatim.

### Analysis

**Fig. 1:**
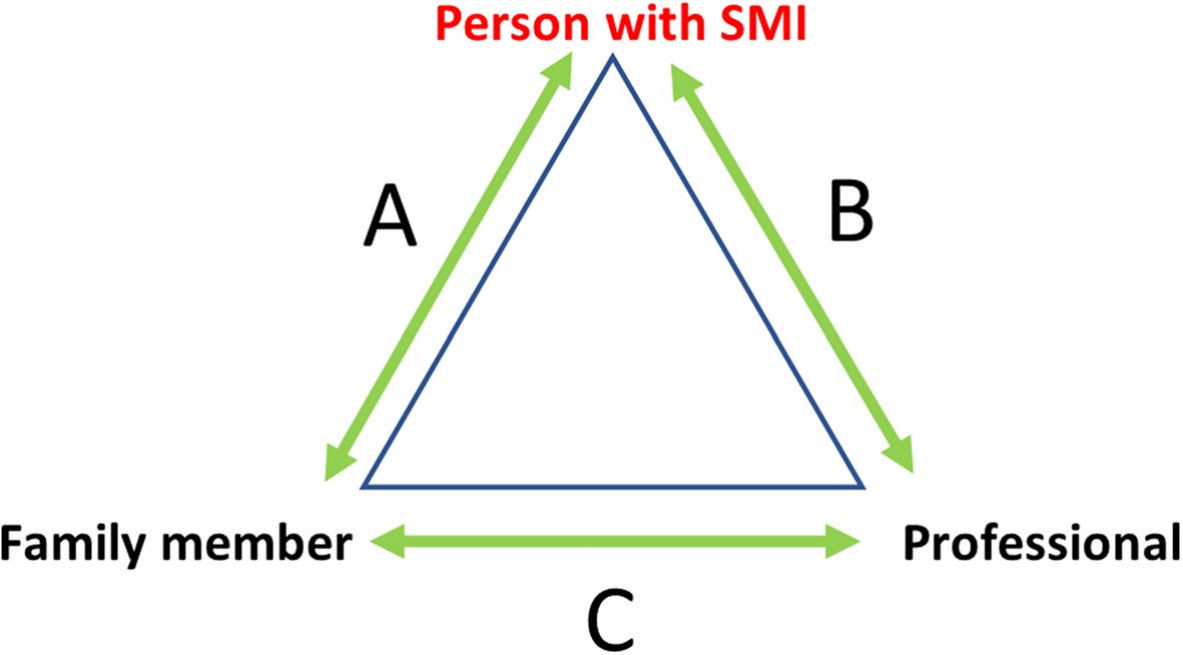
Relationships respondents talk about that themes can refer to. Legend: Example for a respondent with severe mental illness: red font. Relationship talked about: green arrows. Side A: person with severe mental illness talks about relationship with family member. Side B: person with severe mentail illness talks about relationship with professional. Side C: person wil severe mental illness talks about relationship between family member and professional

The nature of our work was explorative, so we conducted an inductive reflexive thematic analysis according to Braun and Clarke [25, 26]. We aimed to develop themes and their interrelation describing the experienced collaboration between triad members, taken from the different perspectives of persons with severe mental illness, family and professionals. Analysis took place at the level of individual accounts, so no comparisons between accounts of related individuals (e.g. a person with SMI and their network member) were made [34]. Developed themes referred to respondents’ views on themselves and others, and either the relationship between themselves and another member of the triad, or between the other two members of the triad, as visualized in Fig. 1.

Our analysis was at a semantic level, and we took a critical realist position. Since the individual interviews were conducted by multiple interviewers, we drew on the Qualitative Analysis Guide of Leuven (QUAGOL) to organize the first stage of analysis in a team process and enhance interpretative depth [35]. In short, two members of the research team per interview (KW, MdK, MLr or TB) read, reread and annotated the interview transcript. They compiled a narrative report and consequently, a point-by-point scheme containing preliminary themes developed from the interview. In group meetings, we then collated the themes seperately for persons with severe mental illness, family and professionals, which TB and MdK then coded back onto the transcripts of the according perspective using MAXQDA software for qualitative data analysis [36]. In the second stage, TB further developed themes and articulated their meaning by assessing their fit across cases, splitting, or merging themes. In the third stage, we used focus group transcripts to enhance our understanding of the main themes developed from the interviews. During each stage, we switched back and forth between the three perspectives, evaluating how themes developed from one perspective were represented in others.

Reflexivity of the researchers, and interpretative depth of the analysis have been influenced by (professional) background of the ones doing the analysis and cross-perspective exchanges throughout data-acquisition, analysis and writing. The mental health professionals (psychiatrist – MdK, psychologist – MLr, resident in psychiatry – TB) had clinical experience within long-term mental healthcare, which helped understanding the care context of the respondents, whereas the outsider perspective of the health scientist (KW) helped to reflect on our view as professionals. Our exchanges with co-interviewers and consultations with the project group on the preliminary results after the interview stage and while drafting the manuscript helped our sensitivity to topics important to persons with severe mental illness and their family. Our analysis thus represents an insider perspective of researchers familiar to a professional point of view, informed by the other perspectives present within the triad, enhancing confirmability.

### Sample

We conducted 52 semi-structured individual interviews: 28 with persons with severe mental illness in long-term mental healthcare (10 of whom in a long-term inpatient setting), 10 with family members and 14 with professionals. Four homogeneous focus groups included persons with severe mental illness receiving care from assertive community based services (*n* = 5) and inpatient services (*n* = 6) respectively, one included family members (*n* = 4) and one included professionals (*n* = 6). The mixed focus group included two persons with severe mental illness, two family members and two professionals. Interviews lasted a mean 71 min (range 23–195) for people with severe mental illness; 86 min for family (range 58–101) and 75 min for professionals (range 48–92). Focus groups lasted about two hours each. To illustrate the general profile of our sample, Table 1 lists sociodemographic characteristics recorded from all unique study participants (interviews and focus groups combined), and clinical characteristics from people with severe mental illness taken afterwards from case files with permission. Persons with severe mental illness in our sample were middle aged on average, mostly had attained a low educational level and mostly were on benefits. Half had regular daytime activities outside their household, and a sizable proportion of the sample lived in a supported housing or inpatient rehabilitation unit. The average illness duration was 25 years, and most had a history of psychosis with multiple episodes or unremitting illness. Most had a primary DSM-5 diagnosis of a Schizophrenia spectrum disorder and about half had a (comorbid) substance use disorder outside smoking [37]. Family and professionals in our sample were heterogeneous in their role and duration of involvement in care.

**Table 1:** Demographic and clinical characteristics of study participants

	Persons with SMI (*n* = 39^a^)		Family (*n* = 14^b^)		Professionals (*n* = 21^c^)	
	N/median^1^	%/range^1^	N/median^1^	%/range^1^	N/median^1^	%/range^1^
Age (years)^*,2^	49	34–65	54	33–77	54	25–68
Sex						
- male	21	54	6	43	8	38
- female	18	46	8	57	13	62
Highest level of education attained^2,3^						
- low	25	64	4	29	3	14
- medium	10	26	6	43	5	24
- high	4	10	4	29	12	57
Housing status						
- Living alone	15	39	5	36	6	29
- Living with family	6	15	9	64	15	71
- Sheltered housing / assisted living	4	10				
- Long-term inpatient rehabilitation unit	14	36				
Relationship status^*^						
- alone	30	77	6	43	6	29
- partner	9	23	8	57	15	71
Place of birth						
- Netherlands	26	71	12	86	18	86
- Other	8	29	2	14	3	14
Regular daytime activities						
- regular or sheltered employment	3	8				
- volunteer work	7	18				
- supported/sheltered day activities	14	34				
- none	15	39				
Primary income						
- wage from job	2	5				
- jobless benefit	13	33				
- disability benefit	23	59				
- other	1	3				
Financial management						
- independent	12	31				
- voluntary assistance	6	15				
- administrator	21	54				
Illness duration	26	8–49				
History of psychosis (yes)	32	82				
Current Primary diagnosis^4^						
- Schizophrenia spectrum disorder	24	62				
- Mood disorders (Bipolar disorder, Major Depressive Disorder)	6	15				
- Personality Disorder	4	10				
- Developmental Disorder	2	5				
- Other	3	8				
Current Substance Abuse disorder^4,5^	16	44				
Clinical staging of respondents with psychosis^2,6^						
- no known history of psychosis	6	19				
- full clinical remission between psychotic episodes	8	25				
- incomplete remission between psychotic episodes	15	47				
- persistent unremitting illness	3	9				
GAF^*^	45	30–60				
Years involved in care^*2^			30	6–40		
Years of collaboration with current professional^*,2^			3	1–5		
Years of work experience in mental healthcare^*^					8	1–44
profession						
- Mental health nurse					9	43
- Psychologist					3	14
- Psychiatrist					5	24
- Spiritual counsellor, Experience worker, Clinical support worker					4	19

*Legend*: SMI: Severe Mental Illness, Illness duration: measured as time between first contact with mental health services and study inclusion, ^a^*n* = 28 interview respondents and *n* = 11 focus group participants, ^b^*n* = 10 interview respondents and *n* = 4 focus group participants, ^c^*n* = 14 interview respondents and *n* = 5 focus group participants, ^1^Median and Range (min–max) reported for continuous measures, which are marked with asterisk^*^, ^2^Missing data. Age: *n* = 1 (SMI) *n* = 3 (family) *n* = 1 (professionals), education level (family) *n* = 1, Clinical staging *n* = 6, family years involved in care: *n* = 4, family years of collaboration with current professional. *n* = 8, ^3^Following ISCED 2011 criteria [38], ^4^Based on DSM-5 diagnosis in case file, ^5^Including gambling disorder in two respondents. Excluding nicotine use, ^6^Following DSM-5 criteria for schizophrenia spectrum disorders [37] ^*^continuous measure

## Results

**Fig. 2:**
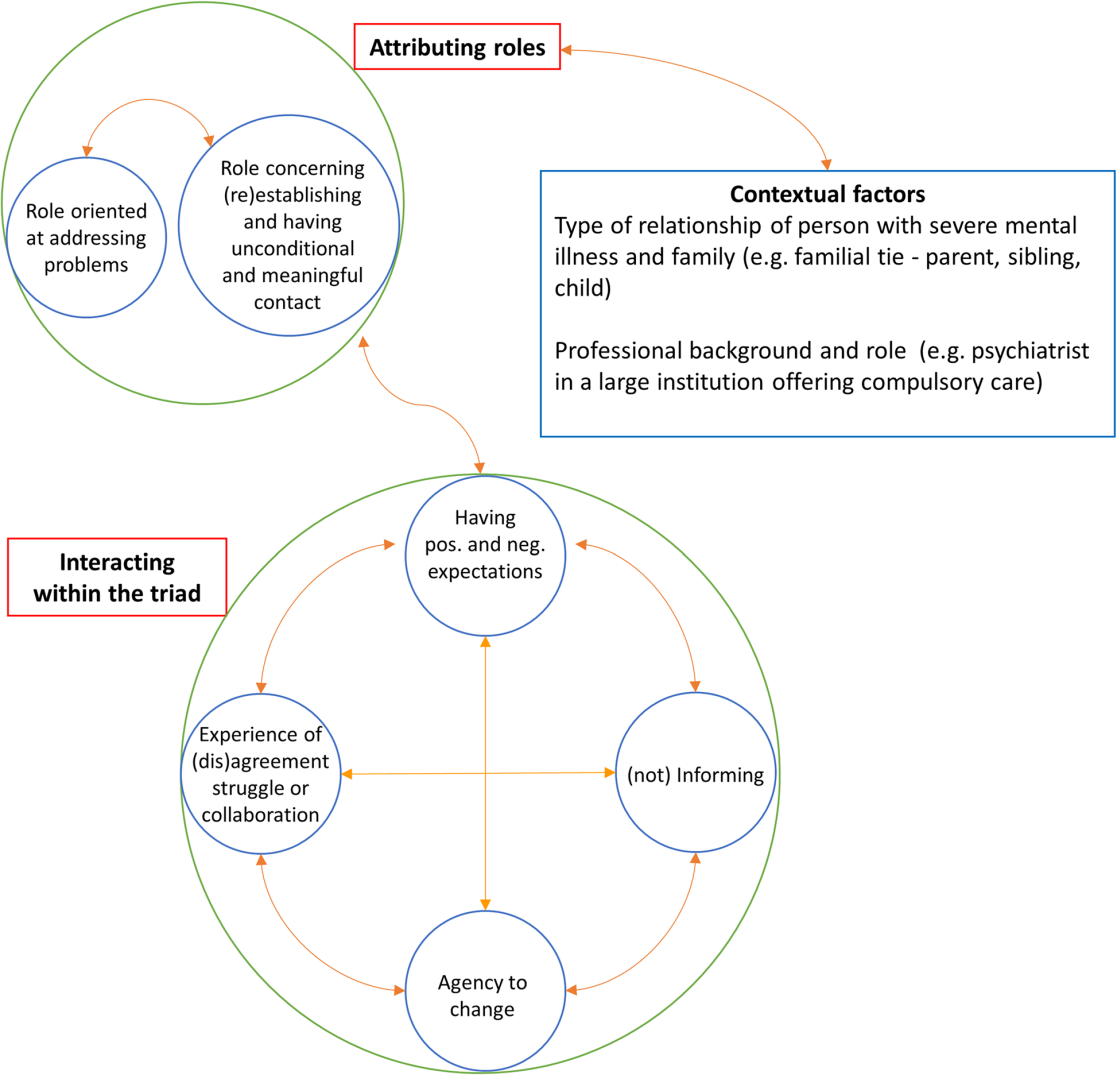
Graphic overview of themes, subthemes and their interrelation. Legend: Main themes: in red boxes attached to green circles containing their subthemes. Subthemes: blue circles. Interrelations between (sub)themes: orange arrows. Contextual factors that influence role attribution: blue box

From the stories of persons with severe mental illness, family and professionals on their (perspective on the) recovery process, we developed two main themes, and introduced subthemes to clarify their content. They represent what a person with severe mental illness, family member or professional does in the triad during collaboration for recovery: (1) attributing roles to members of the triad, which had two mutually influential parts (subthemes): (1.1) an “unconditional” part and (1.2) a “problem oriented” part that included the experience of (mis)alignment over “problem oriented” role; (2) interacting with the others in the triad during the process of recovery, which we describe as a process with four elements (subthemes) which influence each other, not necessarily in a fixed order*:* (2.1) “Having expectations”, (2.2) “(not) Informing”, (2.3) “Taking/Attributing agency (to change)” and (2.4)“Experiencing (dis)agreement, collaboration or struggle”*.* Attribution of roles influenced how interaction was perceived, and vice versa. Figure 2 shows a graphic representation of these processes, and show contextual factors influencing collaboration.

We elaborate on the main themes and subthemes in separate sections below. In the running text, we mainly used quotes that illustrate the experience of diverging perspectives on problem and role and the resulting interaction, to focus on the challenge of collaboration. We listed additional illustrative quotes from all three perspectives in Tables 2 and 3, illustrating the experience of alignment over roles, and positive interaction.

**Table 2:** Theme 1: Attributing roles to members of the triad

**1.1 Role concerning (re)-establishing and having unconditional and meaningful contact**	
persons with Severe mental illness	*"Sometimes he also said, you can call me day and night. Not that I would call him in the middle of the night, but the idea is nice". ****Person with severe mental illness valuing unconditional support***
	*I: "What does it mean to you that your mother is so deeply involved?"*
	*R: "She is my safety. She always got my back. When I had to be hospitalized my mother was there with my youngest sister. And what touched me deeply is that they both said how loving I was and how nice I was and all those kind of things." ****Respondent on the importance of being valued on a difficult occasion***
family	*I: "What do you think is important to your relationship in the future?"*
	*R: [Thinks for a while] "That we can just be brother and sister for a very long time instead of caregiver—patient. […] just that we have a good time watching television together [laughs] Just the simple things".* ***Sibling of a person with SMI on having meaningful contact***
	*"I always bring her a present. And when she sees me, she says, "Oh, no, not again!" Because she doesn't like to talk and I say, "Sorry, I'm only bringing a present." [laughs] And I leave again. […] But then she recovers and says, "oh yeah, thank you." And then quickly closes the door. But somewhere deep down I feel: there still is love in that little moment."* ***Family member on meaningful contact***
	*"I feel that she's doing her job, but also she's doing, that's my impression of her… [stammering] with her heart."* ***Family member, on the way a professional takes up her role toward her father***
professionals	*"I took this to the interview to show to you. I is a farewell present I got from her […] She has written a message on it, that I was always there anyway. Even when things were very bad for her."* ***Professional learned how the person with SMI experienced her care as unconditional***
	*"I can't stand being called a liar and cheater [sighs loudly]. I have to remind myself then, I am dealing with people with an illness here, because I sometimes feel like biting their head off, you know. [laughs] Hello there! I'm human too." ****Professional on feeling personally denounced, and having trouble to remain in a problem oriented role***
**1.2 Role oriented at addressing problems**	
persons with severe mental illness	*"When I had the second collapse and I had the second admission here. Then it became pretty clear to me, I'm not going to live without medication, it's too dangerous." ****Person with severe mental illness attributing the role of patient to himself***
	*Moderator: "So do your social worker and your partner also work together?"*
	*Participant: "She is present at every treatment. Because I also sometimes conceal things. Like attempts to end it, which I’ve done several times"* ***Person with mental illness on role of his partner, from focus group***
	*Participant 1: "How do you like all this support you get?"*
	*Participant 2: "Well, all that attention, in a way it is a burden on you. It takes time, and it does something to your self-image. You do want to be seen as a full human being."*
	*Participant 1: "I recognize that too, yes."*
	*Participant 2: "It’s quite difficult I find, to keep seeing myself as normal. And not just as a patient."* ***Focus group with persons with severe mental illness, on struggling with patient role***
family	*R: "[that I] as brother lost influence on my brother, because he was no longer himself, he wouldn’t listen like normal. A normal conversation had become impossible. I remember that from one of those first times." ****Family member on the moment interaction changed and he lost his role as an advisor to his sibling with Severe mental illness***
	*"My father comes over to my place almost every day. And when he does, he is totally inactive. He sits and he sits. But expects me to make his cup of tea, which he could just make himself. Things like that… And to be honest, it’s too much for me sometimes. But I don't say anything about it, because then I’m like: what else should this man do, who else does he have?"* ***Daughter accepting more because of her father’s vulnerability***
	*R: "Everything was always discussed with me. From top to bottom. And I always offered my opinion. And that was always listened to very carefully. But I also valued listening to the medical knowledge available. Of course I have nothing to say about that."* ***Family member and legal representative explicitizing own expertise/role and professional role***
professionals	*"I find it more important that someone asks me, than how they ask me. Some are not able to ask in a friendly way or formulate it clearly. Sometimes you have to see the real question through the swearing and the desperation. And in the meantime I try to keep contacting their network and see where I can provide support. For example, I keep in touch with the assisted living facility where a patient is staying to see how they are doing." ****Professional accepting difficult behavior based on attributed patient role, and on keeping in touch with the network***
	*"People show one side of themselves in the consultation room. Sometimes I think it's the disordered side. By bringing in the network, you may get to see the healthy side again."* ***Professional seeing someone in a particular role within the consultation room and the role she attributes to family in this matter***
	*"To my regret, we don’t get time to see the network. the main focus of the spiritual counsellor [in our institution] is the service user and supporting professionals, while I also know that, for a service user, his network is the most important. But we spiritual counsellors are not at the gate so to speak. Which is why we don't see the network very often."* ***Spiritual counsellor on his role as defined by his employer***

*Legend*: this table provides additional illustrative quotes for main theme 1 (attributing roles to members of the triad). The quotes are ordered by subtheme (1.1, 1.2) and then by perspective (persons with severe mental illness, family and professionals). A caption in bold directly after the quote provides brief clarifying information on the quote

**Table 3:** Theme 2: Interacting with others in the triad

**2.1 Having positive and negative expectations**	
persons with Severe mental illness	*"I broke off contact, because it wasn't going so well, and I didn’t want them to be disappointed in me. That they would think: he is hospitalized, he is not doing well, he's a little crazy." ****Person with severe mental illness basing his actions on negative expectations***
family	*Family member: "Our dad has been in-and-out of hospital and he may die soon. And the biggest impact is with my sister, I know she worries about him. She says she discusses it with her social worker. But I only get updated on the medication, and I don’t know what is best to do when it will happen, prescribe a higher dosage?"*
	*Moderator: :Do you discuss the situation of your father with your sister?"*
	*Family member: "well, we keep sensitive information from her. We don't know how she will deal with that. We do know that she may start crying over small stuff. So, when dad goes to hospital, then we usually say it's not so bad, that he will be fine."*
	*Person with severe mental illness: "so basically to spare her! But then the blow might come even harder, when it comes."*
	*Moderator: "are you afraid of her reaction?"*
	*Family member: "To get put through the mill again. If she's not doing well, she keeps calling everyone, also at night. To the point we get drained. Everyone gets a roasting."* ***Mixed focus group: exchange on expectations, not informing, not knowing who should do what***
	*"I hope she will accept having an illness to some extent. Maybe her treatment team should teach her some illness insight. Because she thinks: there's nothing wrong with me."* ***Family member has got hopes and expectations on the other triad members***
professionals	*"Sometimes I also find it quite difficult to ask the network. I think, [they] already have quite a burden on their shoulders in terms of family care." ****Professional, voicing her expectation on family burden***
	*"That father was so angry, I found him almost aggressive, also referring to his son, him not doing his best."* ***Professional who experienced negative expectation of a father of a person with severe mental illness***
**2.2 (not) informing**	
persons with Severe mental illness	*"So, I still drink far too much […] So when I talk to that psychiatrist once a year, I feel different than he is after all. And then I tend to pretend some things are a bit nicer." ****Person with severe mental illness presenting things nicer than they are***
	*I: "what made you share it with your social worker?"*
	*R: "if I didn't talk about it, it might trigger psychosis again, because the stress would rise too high. In my first psychosis […] I couldn't actually express it, you just get all caught up in the psychosis, and then you experience such strange things, it’s impossible to explain […] When I fell in love I was like, I have to talk about it, otherwise it will go wrong. [expressing it] gave a little peace. you get some space again for other feelings"* ***person with severe mental illness choosing to inform professional of falling in love***
	*I: "So your case manager at the time had contact with your parents?"*
	*R: "Yes, would that be a triad? The contact between my case manager and my parents was about agreeing, what was the best thing to do at difficult times."*
	*I: "And what do you think about that?"*
	*R: "Uhm, we had agreed on that."* ***Respondent on knowing about professional and his network being in touch***
family	*R: "You know, the social worker doesn't know that either, I shield him a bit. When she calls me again to ask, "Is your father home?" Then I usually say, "I don't know." While I do know that he is at home." ****Family member choosing not to inform professional***
	*"Those are difficult moments, when another new doctor temporarily takes up the case. Those files are very bulky, so you can't expect someone to master that file completely at once either. But it's hopeless for family members, let alone patients, to explain every time what happened and where you came from. And it was very pleasant that his psychiatrist remained a stable factor [for years]."* ***Family member on having to inform professionals new to the case again***
professionals	*"You know, that man who lies in bed so much and talks little. His sister said, he loves to read, ask him to read something to you. And that was a very good tip. Because you get him talking, and he gets out of bed. Yeah, so that was kind of nice." ****professional gets informed by family member***
	*"Once, I treated a man at my ward, put him on medication and he went home and after a week he stopped taking his pills. “why did you stop”, I asked? He said: “Well my mother said, why did you take those pills.” Then I thought: I messed up! Didn’t involve family, gave them no psycho-education.”* ***Professional learned how not informing family impacted outcome***
	*"He also doesn't dare tell his family what exactly he is afraid of. He doesn't want people to worry too much about him. So I think it is important he has a place with me to be honest about his fears."* ***Professional knowing that person with severe mental illness does not inform family***
**2.3 Agency to change**	
persons with Severe mental illness	*R: "Then, I got that mania. And now I am unable to manage my classes, because I am unable to concentrate. So I discuss this with my mental health practitioner: ‘Can we taper off my medication it a little quicker than we agreed?' […] And I don't bother when she says: ‘no, that's not wise, take it easy.’ Then I say: ‘okay’, so it becomes a bit of a consultation."* ***Person with severe mental illness heeding advice of mental health practitioner***
family	*"So he went missing and I spent a year and a half looking for him. Finally, I managed to find him again far away abroad, and managed to arrange for him to fly back." ****Family member going great lengths to get person with severe mental illness home***
	*"One time I did like was when the nurse at the ward said to her: "Mrs. D, you should pour your brother coffee". And my sister immediately took to the task. She had never done that before, pouring coffee for us and stuff. And that's nice because this nurse really helped the contact by suggesting that."* ***Family member on the help that a nurse provided to facilitate contact***
professionals	*"This man with intellectual disability and anxiety issues, he calls his whole family nonstop that he's going to end it. His whole family is going crazy. And so they phone us again: do something. And we are busy finding the right care, but we cannot just keep him from doing that." ****Professional on not having agency, while the network expects otherwise***
	*"So I had given him space to experiment with what he wanted. That wasn't much at first. You can say: you have to do this and you have to do that, but often that makes you lose someone. Many people need their own pace to be able to change."* ***Professional on taking time and leaving agency with the person going through recovery***
**2.4 experience of (dis)agreement, struggle or collaboration**	
persons with Severe mental illness	*"I say to this work supervisor at the day activity center, I want you to stay near me because I am afraid if you leave. And she says:,”ok, that's fine.” But after half an hour I say, I want to go home. And she says: “that's fine. Get in the car and I'll take you home.” Because they also reassured me, my tasks have expanded, so if the girls come for coffee I will bring them coffee." ****Experience of collaboration to develop activities***
family	*"I kept his financial records for him. And in those psychoses, he sometimes would go to my house to take it with him. As a result, he repeatedly lost things and had to reinvent the wheel more or less. At one point, the institution has arranged for a professional financial administrator to put a little less burden on me. And also to keep it clean. Because there were two things running, the money issue and his mental state of course. And that clashed." ****Struggle between a family member and a person with severe mental illness over agency over financial administration, upended by professional taking agency***
professionals	*"The both of us, we see the world differently. I do tell him: I don't have the conviction that you're someone who's going to be killed any minute now. So he knows I see it differently. At the same time, I don't say it's not true what he says, of course that doesn't make much sense. And we do agree that he suffers a lot from the fear he has. So we hope that at some point that fear is going to diminish through treatment. I think that's the reason he keeps visiting, and for feeling safe in conversations, too." ****Professional on differences and common ground for collaboration with a person with a psychosis***
	*"I once had to deal with threatening aggression. A client who had to take medication under supervision because of a court order and we suspected him pretending to take it. And I spoke to him about that. And that made him very angry. Then he held his fist in front of my face. Ready to lash out… That, that was quite impressive."* ***Here, the experience of struggle of a professional amounted to being threatened with aggression***

*Legend*: this table provides additional illustrative quotes for main theme 2 (interacting with other members of the triad). The quotes are ordered by subtheme (2.1, 2.2, 2.3, 2.4) and then by perspective (persons with severe mental illness, family and professionals). A caption in bold directly after the quote provides brief clarifying information on the quote

### Attributing roles within the triad: how establishing contact may enable taking other roles in recovery despite differences over problem definition and vice versa

#### A role concerning unconditional and meaningful contact

The first part of role, concerned (re)establishing and having unconditional and meaningful contact with the other. It was often talked about ardently. Between family and persons with severe mental illness, it would be considered a goal in itself. For family and professionals, having this kind of contact was considered also as an entry to take a problem oriented role. Put otherwise, both parts are mutually influential terms: attributing to someone the role of having a severe mental illness, could mean accepting more aberrant behaviour while keeping up meaningful contact, and having meaningful contact could result in enabling someone taking a problem oriented role. The latter especially occurred when they experienced diverging perspectives on what is going on, see quote A below.

Quote A: Respondent is a family member (RF1), who maintains a close relationship to his brother B (a person with severe mental illness). He drew a line when B had started to break furniture, after they had disagreed earlier that day, about the question if B was doing well.

"At 1 a.m. I heard a huge bang. And then I walked into the living room where I saw him and my television, which had been knocked over. He said he hadn't done anything, but then I was like, now you're going too far for me at my house, even if you don't realize it. Then I said, " [Brother] sit down and I now want you to listen to me: take this emergency medication. And he did take it right away. So fortunately, he somehow was aware that it was ok to follow my demand."

Respondents from all parties described establishing meaningful contact based on (basic) human (emotional) needs would take time. Especially changes in professionals involved were dreaded, by persons with severe mental illness and their family. Professionals in turn reflected on what they were (not) allowed to do as a professional, in other words, on boundaries of their role. They noted this was influenced by the institutional context they were in, see quote B below. Similarly, some persons with severe mental illness and their family reflected on how their original relationship, for example, being siblings or being just a friend, influenced their role within the triad, referring both to a role directed at meaningful contact or at (mental health) problems.

Quote B: A nurse (RP2) on establishing contact by addressing a need, while also stating she did not consider that part of her job.

"With some people you need to hang on. With this client it took me at least a year before I got the idea that we had a bit of a trusting relationship. And that was purely because I cut his hair and shaved his beard, whereas that is not my job at all."

#### A role oriented at addressing problems

The second part of attributing roles referred to problem oriented roles parties attributed to one another. It involved matters like: is someone a patient with a mental illness? Does someone have to accept certain difficult behavior because of the problem? Can this family member decide what is good for a person with severe mental illness? Is this a professional with valid expertise for the matter at hand? Referring to problem oriented roles, the three parties frequently touched on misalignment over the question: is there a problem to be addressed, and if so, what exactly is this problem? Specifically, they referred to persons with severe mental illness being reluctant, unable, or (not) wanting, to take on the role of someone needing/accepting the help another person wants to give, either temporarily or permanently, reflecting the differences in view that may exist in severe mental illness, especially psychosis [10, 12, 13]. Misalignment over role would not occur exclusively between persons with severe mental illness and others, but also between family and professionals. Then it would be linked to views on the role of the person with severe mental illness. When misalignment over role was experienced, the consequent interaction could be experienced as challenging, e.g. including negative expectations, not being informed, feeling powerless, and/or the experience of struggle, see quotes C and D.

Quote C: Exchange between a person with severe mental illness (RM3) in a long- term inpatient service who did not see himself as someone with mental problems, and an expert by experience interviewer (I). It illustrates how attributing roles differently, as a result of diverging subjective perspective, might play out similarly between him and professionals from the service offering help.

"I: And do you have any regular activities?"

RM2: "Music, I play bass."

I: "By yourself, or with other people?"

RM2: "Alone."

I2: "Wouldn't you like to play together?"

RM2: "Yeah, I like to jam. Then you get a better effect of the music."

I2: "Do you happen to know [mentions music project in local community]? Might be below your level, but it is for people with mental healthproblems too."

RM2: "I don't have mental health problems."

I2: "Myself, I did… no, I'm not saying you have mental health problems either. I'm just saying what [music project in local community] is…"

RM2: "It’s for people with mental health problems?"

I2: "yeah, but..."

RM2: "yes, but then I'm not going to go there right?"

Quote D: Partner (RF4) of a person with severe mental illness had been in a relationship with D, whose behaviour had turned strange and dangerous. She attributed to D the role of someone with a mental illness (in this case a psychosis), and to herself the role of arranging appropriate care for D. She ended up in disagreement and struggle with both D and professionals. Her disagreement with professionals only got resolved after she got informed on the mismatch between her take on her role, and that of professionals, who did not think she had a part in decision making. She took action to become a legal representative. The more unconditionally meaningful role she felt to D as a partner and only contact may help explain the great lengths she went.

[When A became mentally ill]

"We did briefly take the path of getting a referral for mental health services. Only D did not go to the appointment, because he believed he was fine." […]

[Later, when D was once more sectioned to a mental hospital, after years in which D had frequently shown recurrent dangerous and confused behavior and also had gone missing for a period of time]

"And the psychiatrist again wanted to send him away as soon as possible. And then I really tried all I could to obstruct and fortunately there was a nurse who pointed out to me: you are not married to him, you are not a family member, you can't tell us anything, because legally, you mean nothing to us. And then I figured out how to become a legal representative. Because I'm the only contact of D."

See Table 2 for further illustrative quotes from all three perspectives on role attribution.

### Interacting with the others in the triad during the process of recovery: how negative expectations may evolve into collaboration.

#### Expectations on what might happen and what actors would do

The parties within the triad often described *expectations* at the outset on what might happen and more specifically, on what they themselves and the others would do, connected to the roles they attributed to themselves and the others. Expectations voiced in the interviews could be negativelly influenced by stigmatized views on themselves or other parties, and/or experience, see quote E.

Quote E: Person with a severe mental illness (RM5) who expected to be admitted to a hospital when professional got involved, so, she chose to misinform her daughter, but eventually experienced that her misinforming instead fueled her admission to hospital by professionals, who took over agency.

R: "So my daughter was going to test if I was all confused. But I didn't want her to know that I was confused, so, I lied to her in that psychosis. But she was not going to take it, and instead got even more convinced I… But I just didn't want her to call again and stuff."

Interviewer: "Why didn't you want that?"

R: "Because I did not feel confused enough yet to be admitted […] I was afraid that would happen of course. And my daughter called and asked: 'where are you?' I said: 'I don't know exactly'. I didn't want to say where I was, because they would send over the police to pick me up. Which they eventually did."

#### (Not) Informing, or being informed on the position of triad members

Informing refers to getting to know the position of the other on an issue, but also to inform others of their own position. Furthermore, they may learn what the other two parties do (not do) with each other, for example, a person with SMI knowing that family and professionals will contact each other in a crisis, or a professional knowing that a person with SMI keeps things secret for his family (see Table 3). But misinforming, or not informing may also happen, as illustrated in quote E.

#### Agency to change

*Having agency (to change*) or experiencing others having agency, refers to feeling empowered and/or responsible to act, and to actually take action. Agency could be experienced as shared. Taking agency related to being informed, but also to attributed roles. Especially professionals and family expressed the dilemma over whether to take matters into their hand, which connected to the question wether or not a person with severe mental illness should be viewed incapacitated to decide to a certain extent by illness, see quote F.

Quote F: Psychiatrist (RP6) who prefers shared agency, e.g. with a person with severe mental illness she treated with lithium. However, she also mentions that she sometimes has to take up the struggle, and decide against the will of a patients, because she felt informed on what would happen otherwise. She also mentions the basis for how she would act: her experience of misalignment on wether or not there is a mental health issue that causes problems.

"With [person with severe mental illness], he wants to go off medication. Ok, then we'll do that. I rather have him find out himself: “when we tapered off that lithium, when I got to this or that dosage, that's when I got off balance”. Rather than to keep telling him, you have to use your lithium." [...]

"The most difficult clients, because I really have to be the doctor there, these are the people who really don’t think they are ill at all. And who continue to think so, even though they have been hospitalized for 20 years, and have received compulsory treatment 20 times. And keep blaming the medication. I can take that role, but it's not my favorite one. That I say no,, you have to get that depot, it is compulsory care. No, we are not going to try tablets another time, because we have already done that eight times and it went wrong eight times. Yes, that's an awkward position."

#### Experiencing (dis)agreement, collaboration and struggle

Lastly, *experiencing (dis)agreement, collaboration and struggle* refers to the experience of working together or against each other, and the feeling of agreement (or disagreement and struggle) over what should happen, see quote G.

Quote G: family member (RF7) on her experience of disagreement with professionals on what she can have a say in, which revolved around the role of the person with severe mental illness as perceived by her (he has shortcomings that need to be supported) versus her perception of the professional view (he is a grown man who needs to tend to himself), and she feels she has to step in because of her negative expectations on what professionals will do.

RF7: "[Sigh] with C, I feel like professionals consider you, a family member, a pain in the neck: It's a grown man. As an aunt, what right do you have to interfere or get involved anyway? That's how they deal with it. That's how I experienced it."

I: "And how does that make you feel?"

RF7: "It makes you feel powerless. [and] I see it differently. I see that he has got shortcomings and that's why I get involved, to help him. To supplement those shortcomings a little bit. Yeah, you have to, otherwise… nothing will happen. Because then they think: that family won't come, they won't care anyway. And then they leave those people for what it is."

I: "So you actually have to keep the professionals going?"

RF7: "Yes. In some cases, yes. And especially if the client is passive."

See Table 3 for more illustrative quotes from the perspectives of persons with severe mental illness, family and professionals on all four subthemes, including ones that illustrate positive expectations, mutual information, having (shared) agency and the experience of having common ground (agreement) for collaboration.

## Discussion

In the current study, we explored perspectives of persons with severe mental illness, most of whom had a psychotic disorder, their family and professionals on collaboration during the recovery process. The study was set in the context of long-term mental healthcare and used a participative, bottom up approach, starting from subjective experiences. We used a reflexive thematic analysis of interviews supplemented by focusgroups, to develop themes that represent processes of collaboration for recovery.

The persons with severe mental illness we have studied have a high level of illness chronicity and lasting psychosocial needs [16], which together with the high prevalence of psychotic illness means that recurring or lasting perspective differences are common. The care context of our research, although recovery oriented, still has an illness-based focus. It also involves frequent changes in living environment and professionals involved [27, 30]. In the following, we will discuss our results in this light.

The two central themes we developed concern *role attribution* and *interacting with the others in the triad*. One part of role attribution referred to establishing unconditional and meaningful contact, put otherwise, working on a meaningful relationship, which was viewed as a recovery goal in itself, a familiar finding in recovery literature on persons with severe mental illness as well as their family [1, 12, 13]. It also was viewed as an entry to taking the second, problem oriented, part of attributing roles, and vice versa. We note that the above understanding of roles within the triad aligns to the widely held interactional view that communication has got a relational and a content aspect [39].

Although recovery by nature is a transdiagnostic concept that transcends the illness domain [2, 40], our findings point out that it matters in collaboration for recovery if there is a shared perspective on the problem. All parties of the triad in our study frequently commented on the impact of differences over problem definition, a core feature of psychotic disorders, a common trait in the context of study.

Our analysis shows the importance of the relational aspect of a role. Respondents from all perspectives found establishing unconditional and meaningful contact important. It would help navigating diverging perspectives. They noted this contact took time to establish. The reverse also held: experienced differences over problem definition and problem oriented role seemed to connect with a negative interaction process: negative expectations including stigma, not informing others or not being informed, not experiencing agency to change and experiencing disagreement and struggle, which could in turn negatively impact meaningful contact.

### Ingredients of collaboration for recovery

Our analysis draws together three key elements of collaboration for recovery from severe mental illness in a triad: diverging perspectives, relational aspects of collaboration and process oriented practice. Firstly, we show how diverging perspectives between triad members, that are common in severe mental illness, not the least in psychosis, may challenge the experience of collaboration for recovery (or even recovery itself, since diverging perspectives may challenge connectedness, a central aspect of recovery). In this context, we note that our results show that framing mental difficulties as an illness may cause considerable tension within the triad, reflecting critique on organization of mental healthcare that is predominantly illness based [41, 42], and that building relationships, which takes time, is viewed as an essential entrance to collaboration, when finding common ground on problem definition is a challenge.

Secondly, the respondents in our study from all perspectives emphasized how discontinuity of (professional) care and relationships may challenge building relationships, both as a goal in itself and as a means to effective collaboration. We draw on Muusse et al., who studied care practices in Dutch long-term mental healthcare, to note that the healthcare system context that our study took place in primarily orders care around patient/individual centered case conceptualization and time/objective bound treatment trajectories, instead of building lasting relationships, which results in discontinuity of professional care [30]. Muusse et al. argue that the matter of defining “good care” is not resolved on a level playing field, as the medical, juridical, and burocratic perspectives on “good care” reinforce eachother, suppressing the relational perspective. The latter is present still, but may need more explicit legitimation in the Dutch long-term mental healthcare for severe mental illnesses. This may extend to systems with a comparable ordering [43]. Without this legitimation, team members may feel unsure if a relational approach to care is indeed “good care” or “part of their job”. Implementing treatment paradigms that prioritize relational practice indeed have faced organizational challenges, both at the health system and health service level [44, 45].

Thirdly, our results foreground the importance of process oriented practice, that is, taking time to make explicit every party’s take on what is going on, and expectations on goals, roles and agency (e.g. who needs what, who can decide). This re-emphasizes the views on care planning of service users and family: it is not the derivation of a care plan that matters most to them, but the quality of the process that leads to it [46]. Models that describe ways to process oriented practice already exist: we point to shared decision making and systemic practices geared at severe mental illness [47–50]. Our results highlight aspects of the process specifically important to recovery-oriented long-term mental healthcare for persons with severe mental illness, where perspectives often diverge: to make explicit in what role(s) parties are involved, question if they are the right persons and to facilitate development of a shared (if not converging) understanding of the problem. Since recovery is a process of change, we suggest regularly revisiting these matters in long-term care.

### Promoting collaboration for recovery in clinical practice

How to promote relational and process oriented care in clinical practice? We point to two lines of work. Both are ways to organize a service rather than a set intervention. Firstly, our results on collaboration for recovery align to open dialogue principles, including its social network approach, psychological continuity and process oriented practice [49, 51]. A large open dialogue trial currently underway in the United Kingdom and implementations in Italy may clarify its feasibility and effectiveness for first episode psychosis, and strengthen the case to investigate such approaches for long-term mental health care [52, 53]. Secondly, care models that set standards for organization of care, team structure and housing facilities such as the Dutch “Active Recovery Triad” model, may work as a scaffold to promote process oriented and relational work [54].

### Strengths, limitations and future research

Strengths of this study are that we used a well defined methodology, importantly emphasizing rich data and analysis through participatory process. This was vital to balance the voices of all parties involved in the triad throughout our research.

The sampling of our study was limited by its confinedness to one institution, and the willingness of participants to share their story. Since recruitment of persons with severe mental illness was via professionals, we likely excluded the most aversive views on professional involvement, although aversion to professional help was still represented in our reseach.

A further limitation of this study is that it presented an analysis of the views of the three parties involved, but did not focus on convergences and differences in views *between actors within a single triad on their collaborative process.* We also did not witness actual interaction between actors *within the same triad.* Our study included “snapshot” views of persons with SMI, family and professionals, but did not map what actually happens *during* a long term collaboration that advances recovery (a series of “snapshots”, or a “movie”). We suggest longitudinal, within-triad research, possibly including ethnographic approaches and/or realist evaluation, to address these matters, and ultimately shed light on what works for whom under what circumstances [34, 55–57]. We finally note that in such research, it will be important to conceptualize families as polyphonic networks including multiple family members, who may have differential perspectives and roles towards a person with severe mental illness.

## Conclusions

This study emphasizes the importance of knowing and valuing diverging perspectives when working in a triad during recovery of severe mental illness, especially psychosis. It proposes processes of collaboration for recovery, including relational and problem oriented role attribution, which influence interaction within the triad. Our study foregrounds the importance of relationally centered and process oriented practice to support the recovery process when perspectives diverge.

## Supplementary Information

Additional file 1. Topic guide.

## Data Availability

The data used and analyzed in this study are not publicly available, as these datasets contain information that could compromise participants’ privacy. The data supporting our findings is available from the corresponding author on reasonable request.

## Abbreviations

Family: Persons relating to person with severe mental illness from social networks (family, friends, important others)
Professionals: Persons relating to person with severe mental illness from professional networks (involving an array of mental health professionals)
SMI: Severe mental illness
