# Supplementary material for: Perspective matters in recovery: the views of persons with severe mental illness, family and mental health professionals on collaboration during recovery, a qualitative study
Source: BMC Psychiatry. 2024 Nov 14;24:802. doi: 10.1186/s12888-024-06198-w (PMC11566249; doi:10.1186/s12888-024-06198-w)
Supplement: Supplementary file 1 — Additional file 1. Topic guide. [file 12888_2024_6198_MOESM1_ESM.pdf]

Perspective matters in Recovery

Supplement 1: Topic list, service user version

Preliminary definitions

Recovery: Four forms or aspects of recovery have been distinguished in the literature: clinical (or symptomatic) recovery, functional recovery, social recovery and personal recovery. These four forms of recovery are interrelated and influence each other. This research focuses on social and personal recovery.

Personal recovery: personal and unique process of giving meaning to that which happened in the past and regaining control of one's life/developing new meaning and goals. Leamy et al. (2011) identified a number of processes important in personal recovery: connection, hope, identity, meaning and empowerment (acronym CHIME). In the topic list, the topic recovery refers to this personal process. Other forms of recovery (e.g., social recovery) may be part of this process for an individual.

Social recovery: improvement of the individual's position in terms of housing, work, income and social relationships. Absent from the Topic List, because may- or may not be contained in the client's personal recovery.

Triad: literally: a group consisting of three parties. In our mental health context: the group of (1) service user, (2) family/friends/colleagues or other loved ones, (3) mental health professional(s) involved (various disciplines); specifically the partnership between them.

Introduction

The purpose of the introduction is to introduce the main topic (recovery).

Discuss form of address during this introduction, adapt your introduction accordingly.

"In this research we interview clients who are in treatment at Mentrum, loved ones and treatment providers about their experiences with recovery.

In the interview we invite you to tell how you came into contact with mental health care (psychiatry) and about your experiences after this.

The purpose of this research is to improve recovery-supportive care for and by clients, loved ones and treatment providers. Within our institution, but also outside our institution by sharing our research results.

Perspective matters in Recovery

Topic list for interview with clients
The purpose of the interview is to get from what has happened to client to client's wishes and desires and helping and hindering factors. In this way the client's recovery is highlighted.

We take the first moment that the respondent came into contact with the mental health service as a starting point and strive to talk about concrete experiences (the recovery story); we use 3 questions propagated by Jim van Os as a framework.

Further questions are asked when aspects come up that address triadic aspects of recovery

A. <u>Questions about recovery</u>

1. What happened

- At a certain point in your life, you came to use mental health services. What was the trigger? What had happened (to you)?

- You are treated at (name of treatment setting). What was the trigger? What happened (to you)?

2. Where do you want to go: wishes and desires

- What ideas did you have (back then) about the future?
- What do you find valuable/important in life?
- Where do you want to go? / What do you hope for? / What makes you happy?
- What are your wishes and desires?
- What is your dream in 1 year (then 5 years)?
- What do you have influence on? (or grip on?)
- Do you manage to shape the things you find valuable/important?

3. What do you need to achieve your goals?
Note: this topic also includes vulnerability and resilience.

- What are you good at / what is your strength?
- What are you proud of?
- How do you make sure you stand tall?
- What is the last thing you accomplished?
    - What or who helped you achieve this?
- What or who has helped you so far/ to shape your desires/ desires?
    - Who believes in you?
    - Who helps you? Who is kind to you?
- What or who has hindered you so far/ to shape your wishes/ desires?
- Have you ever felt very misunderstood?

B. Topics within the recovery narrative

We examine the topic of triadic recovery within the context of the recovery narrative.

It is important to recognize possible subjects related to the topic. Depending on the story that emerges through part A you can determine which subjects within the topics will be explored through questioning.

Possible subjects are listed below. These are by no means exhaustive lists. They are meant to stimulate the sensitivity of the interviewer for the topics within the recovery story.

When questioning about the topics below, it is important to realize that the term "triad" is considered jargon. So (preferably) do not use this term in the interviews.

Triadic recovery

- Wishes/needs that the others in the triad can fulfill
- (Recovery) needs of the others in the triad;
- What you could do for the other in the triad;
- Needs that the triad as a whole has;
- Influence of the organization of care on cooperation in the triad and therefore on recovery
- Influence of culture (broad concept; from culture within the mental health institution to ethnic background)
- Who has got a role in recovery (own role and of others in the triad) - and what is this role?
- Influence of stigma in triad

Tools for talking about recovery
The tools below may be used as needed to start and keep the conversation about recovery going.

Imagery in case people don't get going
Use the picture of sailboat on water with mainland with harbor in background

Tools for maintaining contact

- In the absence of loved ones in the triad: approach the background of this with a detour
- If it does not come up naturally, be sure to ask about clients' successes/strengths within the conversation;
- Mark difficult elements at the end of the conversation; but certainly also the successes / strengths of clients (check that it has not been forgotten!) Give attention to both in the verbal summary.
